# Hospital and Institutional News

**Published:** 1913-10-11

**Authors:** 


### HOSPITAL AND INSTITUTIONAL NEWS.

### BRENTFORD'S INFIRMARY DISCLOSURES: SEQUEL.

The infirmary question at Isleworth remains where it was left a fortnight ago, except that the difficulty of the present position is increased by the fact that the Board now finds itself minus heads to its principal departments—the medical superintendent, the master and matron of the workhouse, and the superintendent and matron of the schools all, we understand, having resigned.

### A PAID TREASURER FOR ST. GEORGE'S HOSPITAL?

In view of the proposed amalgamation between St. George's and Westminster Hospitals we hope there is no solid foundation for the impression which prevails that an attempt is being made to alter the existing constitution of St. George's Hospital by creating the post of paid treasurer and the abolition of the office of medical superintendent. Public opinion and practice have long since caused the discontinuance of the arrangement which formerly prevailed at some of the royal hospitals to pay the treasurer, and we feel convinced that it would not be in the best interests of St. George's Hospital, nor would it tend to help forward the scheme of amalgamation, were the rumoured attempt to be persisted in of creating a paid treasurership at St. George's. We understand there is no one available who has the knowledge and gifts adequately to discharge the duties should such an office be created. Already the amount of money paid in salaries of various kinds at St. George's Hospital is large compared with other similar institutions, and the whole idea is out of date and not calculated to help forward the chief objects to be gained by amalgamating St. George's and Westminster Hospitals. These include the establishment and adequate administration of a modern hospital of the maximum efficiency and usefulness. Further, we are sorry to hear that a good deal of feeling has been excited by some proceedings in connection with the election and retirement of members of the House Committee which are claimed to have been irregular. The urgency of putting an end to any intrigues of the kind, should they exist, is demonstrably proved by the fact that a cleavage has taken place, we understand, between the mover and the seconder of the resolution of the governors agreeing to the sale of the St. George's site and the amalgamation scheme. This is not the time in the history of this hospital to sow dissension or divide the governors. We hope that the latter, or some of the more representative of them, may use their kindly offices to heal the breach, and to make it clear that any attempt to create a new office at a high salary in which to place some one, should he exist, who is anxious to obtain such a position, cannot succeed in the best interests of St. George's Hospital.

### THE "RECOMMEND" SYSTEM AT SHEFFIELD.

The tendency for legacies to diminish under the trend of legislation, in which the forms of direct taxation are increasing, had a direct and forcible instance last week at Sheffield. Mr. Philip K. Wake, chairman of the Board of the Royal Hospital, declared, in lamenting a falling off in legacies and the increasing debt of the institution, that he had learnt from his experience as a solicitor only recently of ten wills in which charitable legacies had been cut down. The Insurance Act was costing the hospital £160 in stamps, and though prescriptions had diminished in the out-patient department, the cost of drugs had increased, so that the Insurance Act had not appreciably benefited the hospital. At the same meeting the Rev. G. H. McNeal urged that greater caution should be exercised in the distribution of "recommends." A large number of people, he said, received treatment at the hospital who could well afford to pay. The Master Cutler, Mr. S. Rossiter Hoyle, supported this view. It has often been shown, in spite of the traditional hold which the "recommend" system has obtained in certain institutions, that any system which tries to thrust on the subscriber the expert responsibility which the admission of only suitable cases to a hospital necessitates is illogical and unsound in principle. The petty patronage which is the essence of the "recommend" system is found often to produce abuse, and this alone is very costly, not only because it cannot wholly be checked under the system, but also because "recommends" are given with reckless disregard to the money equivalent which they bring from individual subscribers. An institution in financial stress should, therefore, address itself to the problem of finding an alternative. Introductory letters, or whatever term may be felt most suitable, which, however, carry with them, in fact, no right or guarantee of acceptance, have been tried with success. Why should Mr. Philip Wake not try a similar expedient at Sheffield?

### MAGISTRATE'S STRICTURE ON A MEDICAL STAFF

The Hon. John de Grey, the magistrate at Lambeth Police Court, has since his appointment become a keen critic of Poor-Law administration. Already he has passed severe strictures on the methods of the Metropolitan Asylums Board in dealing with casuals and with the Camberwell, Southwark, and Lambeth Board of Guardians. No phase of the work is too minute for Mr. de Grey to investigate, apparently with the object in view adversely to criticise the officials. In a recent case in which a man from Gordon Road Workhouse, Camberwell, was charged with refusing to perform a task of wood-sawing, Mr. de Grey had the culprit examined by the doctor at Brixton Prison, who reported that he was not of a robust constitution and had evidence of spinal curvature. In discharging the man Mr. de Grey remarked that he hoped in future that if a man complained he was not strong enough to do the task he would be examined by "a competent doctor."

### SPIRITED PROTEST BY MEDICAL SUPERINTENDENT.

This stigma on the medical staff of the Camberwell Board of Guardians has resulted in a spirited protest from Dr. Keats, the medical superintendent. In a letter he has addressed to Mr. de Grey he points out that since 1907 the man had been an in-and-out inmate of several South London Poor-Law institutions. On September 22 he was examined by Dr. Keats, who found him in an improved condition of health, and he was three times examined by the assistant medical officer, who considered him fit—and this fitness Dr. Keats confirmed—to perform the task of wood-sawing. Dr. Keats added that a lengthy experience of the Poor Law, particularly in connection with cases such as the one under notice, proved it very difficult to get medical men to take the responsibility of certifying such a man as this as being definitely fit. The abuse of Poor-Law institutions, Dr. Keats continued, by loafing malingerers would go on as long as officers, endeavouring to do their duty in the interests of ratepayers, were subjected to unwarrantable criticism by the magistrate who dealt with the cases. He trusted the Guardians would protest strongly against these remarks being made without a full knowledge of the facts. The board decided to support Dr. Keats in his protest, and to inform Mr. de Grey that his remarks were unjustifiable without hearing medical evidence on both sides. Surely a magistrate ought not to be the person to need such a reminder?

### AN ALMONER'S BEQUEST TO ST. THOMAS'S.

In proposing the toast of the hospital and medical school at the old students' dinner of St. Thomas's Hospital Mr. J. Gadesden Wainwright, the treasurer, stated that an old friend of the institution, who had served the office of almoner on many occasions, in the disposition of his property had not forgotten St. Thomas's. A very handsome legacy might be anticipated from him. The almoners had decided to recommend to the governors that the benefaction should be allocated to the out-patient department. But the great difficulty was that of site. "The old friend" is the late respected Mr. C. T. Harris, who acted as almoner at different periods. He was elected a Governor of the Hospital in 1880, a Member of the Grand Committee in 1882, an Almoner and Member of the House Committee in 1900. He has held these positions for various periods up to the date of his death.

### THE PASSING OF THE GENERAL HOSPITAL.

At the annual dinner of the Westminster Hospital Medical School last week, Dr. Purves Stuart cast a glance ahead at the future of the amalgamated institution for which St. George's and Westminster are to unite, and also at that of London hospitals generally. The staffs of the amalgamated hospital, he pointed out, would retain equivalent positions to those which they held at present. He believed the great general hospitals more and more would be deprived of special cases; minor ailments had gone to the panel; tuberculous cases would soon be taken out of their hands; and he thought venereal diseases would follow ultimately. Dr. de Haviland Hall suggested that the United Hospitals of St. George's and Westminster would be a good name for the new institution. Of the sites proposed at Clapham Common and east of Wandsworth Bridge he had come to favour the latter, as both departments could be placed there, but the joint site committee was still sitting. It is curious to note how opinion has see-sawed for and against the special hospital. The fashion in its favour received a check on its multiplicity becoming somewhat reckless, and this check was further aided by the laxity to which a number of small hospitals is liable. The abuse of charity, the cloak given to humbugs, the decentralisation, and the disadvantage of treating certain well-known non-infectious diseases in an institution the staff of which is cut off from that wide experience which a general hospital gives both for the practice and teaching of medicine, all favoured the general hospital, and wisely so. If Dr. Purves Stuart is correct in his prognosis, the "reduced condition" of the general hospital will come about owing to an inevitable and, surely right, tendency for the minor case to be left to the private practitioner, and to the genuinely new specialities like tuberculosis demanding their fitting recognition. But a still growing field in surgery and medicine will yet remain to the general hospitals of the future.

### DOCTOR'S BEQUESTS TO GLASGOW HOSPITALS.

The late Dr. Gavin Paterson Tennent, of Glasgow, formerly honorary consulting physician to the Western Infirmary, Glasgow, has bequeathed £25,000 to the University of Glasgow for such objects in connection with the faculty of medicine as the trustees may decide. The residue of his estate is bequeathed to the Western Infirmary, the Royal Infirmary, the Victoria Infirmary, and the Royal Hospital for Sick Children, Glasgow. A monument of public spirit, the Tennent bequests will remain to show how firmly, in spite of legislative changes affecting both hospital and individual life, those who know it best are convinced of the importance of maintaining the voluntary principle. This instance also should encourage lay testators to devote their charitable bequests to hospitals.

### EXAMINATIONS, EXAMINERS AND THEIR VICTIMS.

The inaugural address at the medical school attached to St. George's Hospital was given this year by Sir William Osler, who chose as his subject "Examinations, Examiners, and Examinees." The atmosphere, he said, in which a medical student now lives is Chinese, not Greek, and too often the one aim is to get through examinations. A medical school, the speaker continued, is a human factor turning out doctors as a finished product after five years. The figures of the General Medical Council showed that the percentage of failures had risen from 12.4 in 1861 to 41.9 in 1895. Twice in the past five years more students had failed in than had passed the final subjects for the M.B. The successful candidates for the F.R.C.S. were the picked steeplechasers of the medical stable. The high-water mark of examination futility was reached in May 1912, when of 118 candidates for the primary Fellowship only thirty-one were approved. He therefore suggested simplifying the curriculum by reducing or abolishing lectures. Subjects that could not be treated in the wards might be dealt with in the *seminar* form. Cunningham's "Anatomy," with its 1,465 pages and very small type, was full of minutiæ, which were a cruelty and had only the Chinese value of a titanic test of memory. The present system of signing up should be changed for one of demonstrator's reports on the character of each student's work. Canada had adopted this system successfully; and in examinations manner should count as much as matter. Evidence of original work should be substituted as far as possible for examinations. Why should not the President and Council of the R.C.S. select each year for the Fellowship fifteen or twenty men who had most distinguished themselves in surgical research? The most grievous mistake of an examiner was to expect from the candidate knowledge of the same quality as his own. One day, no doubt, the General Medical Council would appoint professional examiners to act as assessors to the professors. Medical education should begin with a thorough training in physics, chemistry, and biology, and the student needed more time for quiet study, fewer classes and lectures, and the removal of the incubus of examinations above all.

### AN EFFICIENT TYPE OF MEDICAL REFEREE.

The London Insurance Committee, at a recent meeting, approved of the appointment of six part-time medical referees, up to Christmas of this year, "for the examination of insured persons as to their fitness or otherwise for work in connection with their claims for sickness benefit"—in other words, for the prevention and detection of malingering. The six successful candidates for these posts are distributed widely over the Metropolitan area, each having a section of London allotted to him. The selections, so far as can be judged from the Medical Directory, are all very sound. It is noticeable that practical experience of this kind of work has been the main qualification demanded: only two out of the six are doctors of medicine, but two are police surgeons, one a County Council district surgeon, one a post-office surgeon, and collectively they have seen a great deal of industrial practice in other appointments. Two of the successful candidates, moreover, hold office at the Miller Hospital, one as physician and one as surgeon. Considering the modest remuneration offered (seven and sixpence a case) the Committee have secured an unexpectedly efficient type of referee.

### ACCIDENT TO A DOCTOR AT A NURSING HOME.

A distressing accident at a well-known nursing home not far from Cavendish Square has resulted in the death of Mr. Henry George Walker, one of the senior practitioners of the Kensington district. It appeared in evidence at the inquest that Mr. Walker was at the nursing home with a colleague on professional business, and they were waiting together for the lift, the door of which was ajar. Mr. Walker took a step backwards and fell into the lift well, a distance of some fifteen feet into the basement. His heart was known to be weak, and death resulted from syncope due to cardiac failure, not from any actual gross trauma sustained by the fall. The lift was of the type in which such accidents are mechanically prevented by each door being constructed to open only when the lift is at its level; but the lock had got out of order, and so the device failed. Mr. Walker had practised for a great many years in Marloes Road, on the side opposite to the Kensington Infirmary. He was seventy years of age; qualified as M.R.C.S., L.R.C.P. in 1865; and in the early part of his professional career had been a railway surgeon in South America.

### CHAPLAIN'S THIRTY-ONE YEARS' SERVICE.

Westminster Hospital has during the past week lost one of the oldest members of its staff in the person of the Rev. H. J. Fase, who has resigned after being chaplain for 31 years. Mr. Fase took his degree at Oxford and was ordained in 1870 to the curacy of St. Barnabas, South Kensington, after which he successively served the churches of St. Gabriel, Pimlico, St. Stephen, Rochester Row, Westminster, and Holy Trinity, Westminster, until his appointment in 1882 to the chaplaincy of Westminster Hospital, which became his life work.

### THE WORK OF THE RADIUM INSTITUTE.

When the Radium Institute was opened two years ago a full description of its organisation, equipment and structure appeared in The Hospital. Now that radium has become the favourite thaumaturgus of medicine in the public mind, Sir Frederick Treves, the chairman of the executive committee, and Mr. A. E. Hayward Pinch, F.R.C.S., the medical superintendent, have taken the opportunity to tell the public a few practical results of radium-therapy. The institute possesses 4 grammes of radium, which Sir Frederick Treves stated to be the largest amount owned by any institution within his knowledge. Speaking of radium-therapy, he said that the scientific committee of the institute had proved radium emanations to have the same properties as pure radium, and the committee emphasised that the emanations should be used in treatment. To move the emanations from place to place hollow plates, into which they are pumped, and fixed by liquid air, have been invented. Emanations are stored also in tubes. Thus doctors at a distance can receive radium emanations at a cost which is negligible when compared with that of radium itself. The radium is not destroyed by giving off emanations, but these lose half their strength in three and a half days. Radium water—that is, water impregnated with radium emanations—is being distributed in a strength of from 1 to 2 milliecures per litre, and, Sir Frederick added, in 40 per cent. of patients suffering from arthritis deformans marked benefit has followed its use. The usual dose is half a pint a day for six days in each of six weeks. Hospital staffs send many poor patients, and about 800 paying and non-paying patients have been treated during the past eleven months. The institute is open from 8 a.m. to midnight, and the three medical men, Mr. Alton, the director of the laboratory, two assistants, and three male nurses work in two shifts. The Radium Institute has peculiar problems of administration from the fact that it has to be closed during one month in the year to give the staff that rest which is the only treatment for the radium burns from which all suffer, while no "locums" can be found, since a special training is necessary. Moreover neither the staff nor the radium itself is insurable. The adjoining property has been purchased with a view to extension, when classes will be held, and a research laboratory be provided. Thanks to its very large endowment the institute is now self-supporting, and is about to become incorporated as a non-profit-making organisation.

### ULTRA-CINEMATOGRAPHY.

The applications of cinematography to physiology were illustrated last week by Professor Stirling in the Medical School of Manchester University. He first presented films showing the protoplasmic movements of animalcules by the dark-ground illumination method, while the circulation of chlorophyll granules in certain water-plants were also seen. The actual growth of protoplasm of slime fungi was shown, but the chief point of interest was the method called ultra-cinematography, which shows successive phases of movement slowed down, by which a bird's flight, or the complicated series of muscular actions which we call the step of a man, or a horse walking, can be seen in detail. The beating of a heart normally and during asphyxia was also shown. The growth of living tissue, preserved in aseptic media after excision, was also shown, cell being added to cell, in a fragment of spleen, in the sight of the audience. Cell fission was also made visible. Ultra-cinematography makes invisible processes visible and thereby easier to follow. Does it thereby conduce to more scientific learning and instruction? When we remember what harm has been done in schools by the short-cut method of "teaching" Euclid by algebraic "solutions" the answer to this question can hardly be given so confidently in the affirmative as Professor Stirling himself appears to believe.

### INSURANCE ACT AND ACTON HOSPITAL.

Interesting views regarding the effect of the National Insurance Act on the Acton Cottage Hospital funds were expressed at last week's meeting of the hospital's authorities. The Rev. G. S. De Sausmarez, rector of Acton, declared that since the Act came into operation some of the subscribers had discontinued their subscriptions, and asked whether it would be possible to show that owing to the new legislation the hospital was really in need of more funds. Mr. T. H. Parsons observed that since the passing of the Act the Hospital Saturday Fund had fallen short by over £2,000. The Rector said he had heard of many people having stopped their subscriptions because of the Act, and the lady collectors had found that what he stated was the case. Many members pointed out that an opinion—quite an erroneous one—had got abroad that the Act was doing work formerly done by the hospital. The committee are wise, if late, in dispelling so distorted an impression.

### NEW ASSISTANT SECRETARY TO SATURDAY FUND.

At the quarterly meeting of the Board of Delegates of the Hospital Saturday Fund, held on Saturday last, Mr. Geo. Watts, formerly engaged at the Mount Vernon Hospital, and recently as confidential clerk to the Fund, was elected assistant organising secretary at a salary of £160 per annum. Mr. Watts is twenty-seven years of age.

### ABSCONDING PHTHISICAL PATIENTS.

The Wandsworth Board of Guardians have had considerable trouble through phthisical patients absconding from the annexes at Tooting Home, and returning under the influence of drink. The men apparently climbed the fence, and often remained away for hours. Acting on the suggestion of the Master, the Guardians have decided that in future any patient absconding and returning to the Home shall be transferred to the workhouse. If the patient declined to be transferred, and takes his discharge, the other institutions are to be notified. It was felt by several Guardians that to send patients to the workhouse would not only be a hardship on the men, but might also seriously affect other inmates in the workhouse. Unfortunately of late there have been several cases of absconding, and it was believed that the only method of dealing effectively with what was becoming a serious menace to the discipline of the Home was to adopt the above proposal.

### METROPOLITAN ASYLUMS BOARD AND SANATORIA.

Ten Metropolitan Borough Councils have approved the Holborn Borough Council's resolution, referring to the resolutions of the London County Council of June 17 as to the arrangements to be made to provide sanatoria and hospital provision for tuberculous patients in London. In effect the Holborn Borough Council favoured the Metropolitan Asylums Board becoming the authority for the institutional treatment of all classes of tuberculous patients, and that the borough councils should be represented on the Board, so that the local sanitary authorities should be more closely related to the hospital authority in London. In the meantime the Local Government Board have informed the Metropolitan Asylums Board that in their opinion the London County Council should be the authority for the treatment of tuberculosis in London, all other agencies being co-ordinated through it, and that much of the accommodation required should be provided by the managers under their now famous agreement with the Council as regards insured persons under Section 39 of the

Insurance Act, and as regards other persons under Section 3 of the Public Health (Prevention and Treatment of Disease) Act, 1913, and Section 80 of the Public Health (London) Act, 1891. Apparently, then, the Metropolitan Asylums Board is to do the work, and, if Mr. Burns' Department has its way, the County Council will be officially and primarily responsible for it.

### DR. ROBERT JONES ON FAILURES.

Among the most interesting opening addresses delivered on Monday night, when 250 new evening institutes recently established by the London County Council were opened, was that of Dr. Robert Jones, the distinguished alienist and medical superintendent of Claybury Mental Hospital. Speaking on "Lessons from Failures," at the Cranbrook Road Institute, Bethnal Green, he said knowledge and culture were the best passports to influence and social standing; but the younger people had to overcome slackness and indifference. A syllabus of classes on a social basis should be established, and manners were greatly needed. From statistics he showed how failures were divided between, and occasioned by, crime, drink, mental disease, and vagrancy, and affirmed that it was character, which was not measured by success, which carried men through the struggles of life. He urged that more concentration and less diffusion of study should be the aim of culture. Such an address gives ample material for disquisition, but we must only note that it has been remarked by someone that "equality is the basis of good manners," which is profound and little understood truth; that character, or, in the philosopher's phrase, will, is indeed the motive power in both life and art, each being valuable in accordance with the degree of fulfilment which this will has in it; and, lastly, that a specialist like Dr. Robert Jones is possibly a little biassed in favour of specialism. It would be a mistake to suppose that by encouraging specialism we do away with the sciolist. The sciolist who calls himself a specialist is the most disastrous of shallow thinkers. The wider the man's studies are the more he learns the unity of knowledge; and though "a liberal education" has produced its full share of shallow thinkers, we believe there is more hope for "culture" in the wide sense than there is for specialism. Specialism can only produce its full fruits when entered upon at the end of a long general education. Otherwise, is lost the plasticity of mind from which alone concentration can escape the academic dry-as-dust and become the valuable thing which it ought to be. A consideration of the late Samuel Butler's work reveals that activity in culture and life are no bad preparation for specialised scientific study and original research.

### A LEGAL POINT FOR PANEL DOCTORS.

Who is responsible when a panel doctor does not attend an insured patient? What may prove a test case, in its first stage, came before the Liverpool County Court last week, when an insured clerk, through his father, sued Dr. John Dunlop, of Anfield, for £2 6s., being the expenses incurred in engaging another doctor through the defendant's inability or refusal to attend the plaintiff, whom he had accepted as a panel patient. As a result of a chill after football, said counsel, Dr. Dunlop was sent for. As he was out a message requesting his attendance was left. He never came, and four hours later promised personally to come, but failed to do so. Evidence to show actual refusal to attend having been called, the defence was begun. Its argument was that under Section 67, Subsection 2, of the Insurance Act it was with the Insurance Committee alone that the plaintiff could deal if a panel doctor were unable to attend him. It was contended, as the fee was payable by the medical service sub-committee, that the Liverpool Insurance Committee and not the defendant was responsible. Judge Thomas gave judgment for the plaintiff, and, remarking on the importance of the points raised, gave leave to appeal on a point of law. Pending its result we refrain from comment.

### TOTAL ABSTAINERS AND MEDICATED WINES.

It has long been recognised how relatively large a proportion of alcohol is contained in many so-called "Temperance" drinks, and Dr. Robert Simpson, of Plymouth, now draws attention to the growing use of medicated wines, by total abstainers as well as by the general public. The art of the medicine seller is to call something which has a pleasant flavour, whether cocoa or an alcoholic drink, medicine, and it will be bought by a public only too glad to flatter its natural appetite for sweetmeats and stimulant by such a title. But such is the loose thinking common among the rank and file of all movements which show a fanatical distaste for any specific habit or custom, that these are as much duped by a scientific sounding title as the general public which is indifferent to everything except fashion. It is just as unwise thoughtlessly to condemn the general use of alcohol as it is to enforce the general use of milk. Individual idiosyncrasies have to be considered and also, as is beginning to be recognised in reference to milk, conditions of "manufacture." The serious point is, as Dr. Simpson pointed out, that numbers of cases of inebriety owe their origin to indulgence in tonic wines, to which as a class, he added, the medical profession is becoming increasingly opposed. Here then is a scientific attitude; it is not the alcohol, *per se*, which the profession objects to, but the ridiculous device by which people prone to alcoholic excess cloak their indulgence under the pious disguise of "taking a tonic." Since all properly digested food is a stimulant, and since overeating produces results medically analogous to overdrinking, it is unwise to make alcohol, or roast beef for the matter of that, the culprit. The true culprits are the men and women, not the things which they eat or drink. Having come to the point of seeing that adulteration is not the less adulteration when called "preservative," cannot at any rate the total abstainers learn that alcohol is still alcohol when called a tonic? Perhaps there was more wisdom than is now believed in the former opinion that the value of a medicine was in proportion to its nastiness.

### CHEAP THERMOMETERS: A WARNING.

A very timely and instructive communication to the *Lancet* by Dr. J. B. Cook, the medical superintendent of St. Giles' Infirmary, Cleveland Street, should serve as a lesson to institutional managers not to buy goods at prices too low to be compatible with good quality. A well-known firm of surgical-instrument makers undertook to supply clinical thermometers for use in the infirmary at 10s. 6d. the dozen—a tender, we may remark, which should never have been accepted by the responsible authorities, presumably the Guardians. Three dozen were delivered, and Dr. Cook (who probably had his doubts about the quality obtainable for such a cut-throat price) tested them by total immersion for five minutes in warm water continuously agitated to ensure its uniform temperature. The thermometers were then read, with the following results: 99.4, 98.2, 99, 101.6, 101, 101.2, 98.8, 99.6, 101.6, 100.6, 99.2, 99, 100, 101.6, 99, 99.6, 98.2, 100, 100.2, 99.8, 98.4, 98.6, 99, 98.6, 99.8, 96.4, 99, 98.2, 100.8, 96.8, 98, 98.6, 101.6, 98.8, 99, 100. The whole batch was returned to the vendors, who replied that they could not guarantee the accuracy of such cheap thermometers, but that for 18s. the dozen they would supply reliable thermometers, guaranteed to one-fifth of a degree. Accordingly, three dozen were ordered and tested as before; but the readings were just as discordant as in the first batch, so these too were rejected. Finally the firm sent another batch in charge of one of their own representatives armed with two thermometers tested at Kew; once more the readings were hopelessly inaccurate and variable, and the maker's representative retired crestfallen. It is unnecessary to point the moral of this very serviceable warning.

### FROM ASSISTANT SURGEON TO CANON.

In a most interesting address on medicine as a liberal education, at the opening of the winter session of the Middlesex Hospital Medical School, Mr. Sampson Handley quoted many of the "renegades" from the profession of medicine who have reached distinction in other walks of life. Most of the names he mentioned, such as Conan Doyle, Charles Wyndham, Robert Bridges, Seymour Haden, Huxley, Keats, St. Luke, and others, are familiar enough in this connection. Dr. Bridges was, as we were all reminded when he became Poet-Laureate, assistant-physician to two London hospitals many years ago. The institutional world, it now appears, can also claim the credit for having trained a distinguished clergyman; for Canon Arnott, now and for twenty-eight years rector of Beckenham, was at one time on the staff of the Middlesex Hospital as assistant-surgeon. Canon Arnott is, needless to say, a Fellow of the Royal College of Surgeons; and, as the author of a work on cancer, he anticipated the special attention which his old school and hospital now pays to that disease. Canon Arnott has absolutely abandoned surgery, and abstains even from giving advice about medical matters; as pastor of a big parish with four churches he has no time to keep in touch with modern developments.

### HOSPITAL SATURDAY FUND.

We are glad to learn officially that our notice last week caused many of the hospital representatives to attend the meeting of the Board of Delegates on the 4th instant, and that after an able and tactful speech from the mover, Mr. Aldrick, the Mayor of Poplar, and considerable discussion, the resolution we published in our issue of the 4th instant was carried by a large majority. The decision depended greatly on the report presented by the Distribution Committee, which set forth all the facts in a colourless and impartial manner, that does those responsible infinite credit. All's well that ends well.

### AN IMPORTANT PHARMACEUTICAL DISCOVERY.

The International Congress of Pharmacy, which has been held at The Hague, was of a highly successful character. It is interesting to note that Professor Bourquelot, who was undoubtedly the greatest personality at the Congress, is chief pharmacist at the Hôpital Laënnec, Paris, as well as a Professor at the Paris School of Pharmacy, and a member of the Committee of Revision of the French Pharmacopœia. His lecture at the closing meeting of the Congress on "The Synthesis of Glucosides by Means of Ferments" was a review of the work on this subject which he and his collaborators have accomplished during the last twelve years or so—work which places him at the head of pharmaceutical researchers. He has recently made the important discovery, which was announced for the first time at the Congress, that the action of ferments on glucosides is not only decomposing, but also synthetic, and that, as the result of this discovery, he had been able to synthesise a whole series of glucosides.

### THIS WEEK'S DRUG MARKET.

Business during the week has not been on a very considerable scale, but there has been a fair amount of inquiry, which may lead to orders in due course. Immediately after last week's report was written the price of glycerine was, as anticipated in the report, advanced by the refiners; although a further advance is not expected immediately, a reduction is not very probable. Citric acid is again rather dearer, and the price of tartaric acid has an upward tendency, while makers have raised their quotations for Rochelle salt and seidlitz powder. Following the announcement of a reduction in the prices of salicylic acid and sodium salicylate the quotation for salol has been slightly reduced. The price of boric acid has been advanced. Cocaine is unchanged in price, but the market is "weak," and a drop in price would not come altogether as a surprise. Santonin has again been advanced in price, notwithstanding the very high figure previously quoted. There is no quotable change in opium values, and a further reduction is hardly expected; both morphine and codeine, however, have a downward tendency.

